# Lifestyle and geographic insights into the distinct gut microbiota in elderly women from two different geographic locations

**DOI:** 10.1186/s40101-016-0121-7

**Published:** 2016-12-12

**Authors:** Ji-Hee Shin, Minju Sim, Joo-Young Lee, Dong-Mi Shin

**Affiliations:** 1 Department of Food and Nutrition, Seoul National University College of Human Ecology, 1, Gwanak-ro, Gwanak-gu, Seoul 08826 Korea; 2 Department of Textiles, Merchandising and Fashion Design, Seoul National University College of Human Ecology, Seoul, Korea; 3 Research Institution of Human Ecology, Seoul National University, Seoul, Korea

**Keywords:** Gut microbiota, Living environment, Lifestyle, Diet, Physical activity

## Abstract

**Background:**

A large number of microorganisms reside within the gastrointestinal tract, especially in the colon, and play important roles in human health and disease. The composition of the human gut microbiota is determined by intrinsic host factors and environmental factors. While investigating environmental factors to promote human health is of great interest, few studies have focused on their effect on the gut microbiota. This study aimed to investigate differences in gut microbiota composition according to lifestyle and geographical area, even in people with similar genetic background.

**Methods:**

We enrolled ten and nine elderly women in their seventies from island and inland areas, respectively. Fecal samples were obtained from individuals, and bacterial 16S ribosomal RNA genes were analyzed by next-generation sequencing to define the gut microbiota composition. We assessed their diet, which can influence the gut microbial community. We also conducted physical examination and determined the physical activity levels of the subjects.

**Results:**

The inland subjects had a significantly higher rectal temperature, systolic blood pressure, and heart rate and a significantly lower physical activity score than the island subjects. Fecal samples from the island group showed a tendency to have greater microbial diversity than those from the inland group. Interestingly, the microbial community composition differed significantly between the two groups. *Catenibacterium* was enriched in subjects from the island area. *Catenibacterium* showed a negative correlation with rectal temperature and a positive correlation with the dietary level of animal fat. In contrast, *Butyricimonas* was enriched in the inland subjects. A positive correlation was found between *Butyricimonas* and mean arterial pressure.

**Conclusions:**

This study identified differences in the gut microbiota composition between elderly women from different parts of South Korea, and our findings suggest that further studies of the human gut microbiota should evaluate aspects of the living environment.

## Background

Microorganisms reside on all human body surfaces, but the majority colonize the intestinal tract, which harbors approximately 10^14^ bacterial cells [1]. The most abundant phyla in the human gut are Firmicutes and Bacteroidetes, which account for more than 80% of the total [2]. Although the human gut microbiota consists of a few dominant phyla, it varies dramatically from individual to individual at the genus and species levels [3]. Furthermore, gut microbiota composition can influence host health and disease. Microbes in the gut are involved in many functions that benefit the host such as vitamin synthesis, digestion of dietary fiber, and regulation of the inflammatory response [4]. Furthermore, communication between the gut microbiota and host brain can affect brain function and behavior.

The composition and diversity of the gut microbiota are determined by host properties such as genetic factors and age and environmental factors such as diet, physical activity, and geography. The host innate immune system derived from genetic disposition can lead to an overrepresentation of particular bacteria, resulting in different makeup of the gut microbiota [5]. In addition, diet has been recognized as a representative environmental factor that influences the gut microbiota composition. For example, animal models of a Western diet showed an increase in the abundance of the phylum Firmicutes and a decrease in that of the phylum Bacteroidetes [6]. A cross-sectional study in humans also showed that enterotype clustering was strongly associated with long-term diet containing a large proportion of protein/fat versus carbohydrates. Moreover, two groups of children from urban Europe, where people eat Western-style diets rich in animal protein/fat, and rural Africa, where people eat high-carbohydrate diets, showed a different gut microbiota composition [7].

Geography also influences gut microbiota composition. A large structural survey of the fecal microbiota of healthy young adults from nine provinces of China reported that the subjects were clustered mainly according to geography and ethnicity [8]. Similarly, a cohort study covering rural areas of Venezuela and Malawi and US metropolitan areas indicated that the dietary pattern of the host influences the type of fecal microbes [9]. In addition to the aforementioned factors, many lifestyle factors such as smoking habit [10], stress [11], and circadian rhythm [12] influence the intestinal microbiome. In a recent study, exercise was highlighted as an important factor for microbial community composition in that athletes had greater gut microbiota diversity than controls [13].

Jeju Island is the largest island of South Korea with a total area of 1848 km^2^ and is located at longitude 126° 08′–126° 58′ E and latitude 36° 06′–33° 00′ N. The proportion of people aged 65 years or over on the island is steadily increasing, most of whom are employed in farming and fishing. In terms of the general infrastructure, most parts of Jeju Province are close to rural areas. Indeed, breath-hold women divers (*haenyeo*) who collect seafood without any respiratory equipment reside mainly on Jeju Island [14]. According to Jeju Special Self-Governing Province, breath-hold women divers are aging, with 43.3% being over 70 years old in 2010. In contrast, Seoul is the largest city in South Korea, in which 22.8% of the economically active population is aged 65 years or over [15]. Accordingly, the elderly who live in urban areas likely have distinct living environments, including in terms of dietary patterns, physical activities, and lifestyle compared with those working as *haenyeo* on Jeju Island. Although the impact of environmental factors on the gut microbiota is thought to be considerable, few studies have determined whether differences in regional characteristics within Korea influence the gut microbiota composition.

The objective of this study was to investigate the general characteristics and environmental factors, such as dietary pattern and physical activities, of two groups of elderly women from Seoul and Jeju Island and to characterize their fecal microbiota composition.

## Methods

### Participants

We enrolled nine and ten individuals older than 65 years from Seoul and Jeju Island, respectively, into the study. Ten Jeju Island dwellers were long-term exposed to cold sea water at 13.6 °C in order to catch the seafood. The average duration of diving was about 50 years. We excluded participants who (1) received antibiotics within 3 months, (2) had a history of a gastrointestinal (GI) tract surgery or disease, and (3) have been taking probiotics.

### Health status assessment

Rectal temperature (RT) was measured every 5 s for 1 h using a data logger (LT-8A; Gram Corporation, Japan) which was inserted 16 cm beyond the anal sphincter. Systolic blood pressure (SBP) was measured three times over the resting arm at the end of the resting period. Heart rate was recorded every 5 s for an hour at rest using a heart rate monitoring device (RS400, Polar Electro Oy, Finland). To estimate resting metabolic rate (RMR), oxygen (O_2_) consumption, carbon dioxide (CO_2_) production, and ventilation were continuously measured for an hour while sitting on a chair (Quark b2, COSMED, Italy). The environmental chamber during the RMR measurement was maintained at a thermal neutral condition (25 ± 0.5 °C in air temperature and 50 ± 5% RH in air relative humidity). Prior to the respiratory measurement, the respirometer was calibrated using room air, a standard gas mixture (4% CO_2_, 16% O_2_, balance nitrogen), and a volume calibration using a 3-l syringe.

### Dietary assessment

Dietary survey was conducted by a 24-h recall method including three random days composed of two weekdays and one weekend day. The 24-h dietary recall interview (24HR) is one of the standardized tools for dietary assessment. Participants were asked to recall the types, amounts, ingredients, and cooking methods of the food that they had taken in for the previous 24 h and the location of the meal. In consideration of the age of participants, face-to-face interviews were selected, and interviewers utilized full-scaled pictures of various food items to assist with participants’ estimation of portion sizes of foods. Intakes of total calories, carbohydrates, proteins, fats, vitamins, and minerals were analyzed by using the Computer Aided Nutritional Analysis Program (CAN-Pro, 2010) 4.0 of the Korean Nutrition Society.

### Quantification of physical activity

The Korean version of International Physical Activity Questionnaire (IPAQ) short form [16] was used to quantify physical activity in the past 7 days, and the investigation was carried out through phone interview individually. Participants were asked to answer the number of days and the number of minutes per day regarding their vigorous, moderate, and walking physical activities. After that, the intensity of physical activities was expressed as metabolic equivalent (MET)-minute per week according to the IPAQ scoring protocol [17]. Three levels of physical activity (high, moderate, low) were used to classify the participants. The following values were used to analyze the IPAQ data: light = 3.3 METs, moderate intensity = 4.0 METs, and vigorous intensity = 8.0 METs [17]. Four continuous scores were calculated by the following formulas:

1 $$ \frac{\mathrm{Vigorous}\ \mathrm{MET} - \mathrm{minutes}}{\mathrm{week}} = 8.0 \times \frac{\mathrm{minutes}\ \mathrm{of}\ \mathrm{activity}}{\mathrm{day}} \times \mathrm{days}\ \mathrm{per}\ \mathrm{week} $$

2 $$ \frac{\mathrm{Moderate}\ \mathrm{MET} - \mathrm{minutes}}{\mathrm{week}} = 4.0 \times \frac{\mathrm{minutes}\ \mathrm{of}\ \mathrm{activity}}{\mathrm{day}} \times \mathrm{days}\ \mathrm{per}\ \mathrm{week} $$

3 $$ \frac{\mathrm{Light}\ \mathrm{MET} - \mathrm{minutes}}{\mathrm{week}} = 3.3 \times \frac{\mathrm{minutes}\ \mathrm{of}\ \mathrm{activity}}{\mathrm{day}} \times \mathrm{days}\ \mathrm{per}\ \mathrm{week} $$

### Sampling and genomic DNA extraction

Four participants from each group were randomly selected for the sequencing analysis. A stool sample was immediately weighed and frozen at −80 °C. Total bacterial DNA was isolated from stool by using the QIAamp^®^ fast DNA Stool Mini Kit (QIAGEN, Hilden, Germany) according to the manufacturer’s instructions with the following additional steps. Briefly, an average of 200-mg stool sample was homogenized with 5-mm sterilized steel bead in ASL buffer using TissueLyser (QIAGEN, Hilden, Germany) for 1 min at 30 Hz. We increased the heating temperature of the fecal lysate at 95 °C to enhance the lysis of the cell walls of gram-positive bacteria. In the last incubation step, we increased the incubation time from 1 to 5 min to increase DNA yield. Extracted genomic DNA was confirmed via gel electrophoresis and was quantified by spectrophotometer NanoDrop ND-2000 (Thermo Scientific, Waltham, MA, USA).

### Amplification of 16S rRNA gene and sequencing

Hypervariable regions (V1–V2) of 16S ribosomal ribonucleic acid (rRNA) gene were amplified using barcoded universal primers for each sample. Sample-specific barcoded primers for each sample are listed in Table 1. Polymerase chain reaction (PCR) was carried out by using BioFact F-Star taq DNA polymerase (BioFACT™, Seoul, Korea). Briefly, a final volume of 50 μl PCR reaction contained about 20 ng DNA template, 5 μl of 10X Taq buffer(20 mM Mg^2+^), 1 μl of 10 mM dNTP mix, 2 μl of forward and reverse barcoded primers (10 pmol/μl), and 0.25 μl of DNA polymerase. PCR reactions were amplified using a GeneAmp^®^ PCR system 9700 (Applied Biosystems, Foster City, CA, USA). The PCR program was as follows: initial for 5 min hold at 94 °C, followed by 28 cycles of denaturation (30 s, 95 °C), annealing (30 s, 60 °C), and extension (30 s, 72 °C), with a final extension step of 10 min at 72 °C followed by holding at 4 °C. The PCR product was confirmed by using 1% agarose gel electrophoresis and visualized under a Gel Doc system (BioRad, Hercules, CA, USA). The amplified products were purified with PureLink Quick Gel Extraction and PCR Purification Combo Kit (Invitrogen, Carlsbad, CA, USA) and quantified by the Qubit 2.0 fluorometer (Invitrogen, Carlsbad, CA, USA). The size of library was assessed by BioAnalyzer (Agilent Technologies, Santa Clara, CA, USA). The library was diluted to 100 pM and emersion PCR and enrichment were performed using Ion Chef™ instrument (Thermo Fisher Scientific, Waltham, MA). The enriched library was loaded in Ion 520™ Chip and sequenced by an Ion S5 XL platform (Thermo Fisher Scientific, Waltham, MA) according to the manufacturer’s instructions by Thermo Fisher Scientific, Inc. (Seoul, Korea).

**Table 1:** List of fusion primer sequences

Name	Sequence (5′-3′)
A-Adaptor	CCATCTCATCCCTGCGTGTCTCCGACTCAG
P1-Adaptor	CCTCTCTATGGGCAGTCGGTGAT
Stuffer	GAT
Fusion barcoded primer (reverse)	
R361	CCTCTCTATGGGCAGTCGGTGATCYIACTGCTGCCTCCCGTAG
Fusion barcoded primer (forward)	
IonXpress_9	CCATCTCATCCCTGCGTGTCTCCGACTCAG**TGAGCGGAAC**GATAGAGTTTGATCMTGGCTCAG
IonXpress_10	CCATCTCATCCCTGCGTGTCTCCGACTCAG**CTGACCGAAC**GATAGAGTTTGATCMTGGCTCAG
IonXpress_11	CCATCTCATCCCTGCGTGTCTCCGACTCAG**TCCTCGAATC**GATAGAGTTTGATCMTGGCTCAG
IonXpress_12	CCATCTCATCCCTGCGTGTCTCCGACTCAG**TAGGTGGTTC**GATAGAGTTTGATCMTGGCTCAG
IonXpress_13	CCATCTCATCCCTGCGTGTCTCCGACTCAG**TCTAACGGAC**GATAGAGTTTGATCMTGGCTCAG
IonXpress_14	CCATCTCATCCCTGCGTGTCTCCGACTCAG**TTGGAGTGTC**GATAGAGTTTGATCMTGGCTCAG
IonXpress_15	CCATCTCATCCCTGCGTGTCTCCGACTCAG**TCTAGAGGTC**GATAGAGTTTGATCMTGGCTCAG
IonXpress_16	CCATCTCATCCCTGCGTGTCTCCGACTCAG**TCTGGATGAC**GATAGAGTTTGATCMTGGCTCAG

Barcode sequences are shown in bold type

### 16S rRNA gene sequencing data analysis

The change of microbial community composition was determined using Ion Reporter™ Software. Briefly, primer sequence and the sequences of shorter reads than 300 bp were excluded. Then, V1–V2 hyper variable regions of 16S rRNA genes are aligned using Curated Micro SEQ(R) 16S Reference Library v2013.1 and Curated Greengenes v13.5 with 90% alignment coverage. Genus and species were identified at a confidence threshold of 97 and 99%, respectively.

### Statistical analysis

All values are expressed as mean ± SD. Statistical significance was evaluated by Mann-Whitney test. Statistical analyses were performed with GraphPad Prism version 6 (GraphPad Software, Inc, La Jolla, CA). Pearson correlation coefficients were conducted by *R* (www.r-project.org). In order to identify differentially abundant bacterial genus between two groups, we used linear discriminant analysis (LDA) effect size (LefSe) analytic method via the Galaxy workflow framework (https://huttenhower.sph.harvard.edu/galaxy/root) [18].

## Results

### General health and geographical characteristics of subjects

The gut microbiota composition is affected by physiological characteristics of the host, such as sex, body weight, antibiotic usage, and genotype. Therefore, we first investigated the physiological characteristics of the subjects (Table 2). No significant differences were found in age, weight, body mass index (BMI), or body surface area (BSA) between the two groups. However, the height of the subjects from the island was significantly higher than that of those from the inland area (*P* = 0.0207). Systolic blood pressure (SBP), resting mean arterial pressure (MAP), and heart rate (HR) were significantly lower in the island group than in the inland group (*P* < 0.05). Interestingly, whereas no differences were observed in skin temperature, the rectal temperature (RT) of the island group was significantly lower than that of the inland group (*P* = 0.0232).

**Table 2:** Health and general characteristics of subjects

Parameters	Inland (*n* = 7–9)	Island (*n* = 8–10)	*P* value*
Age (year)	72.89 ± 4.57	70.00 ± 2.94	0.3747
Height (cm)	151.4 ± 4.13	156.2 ± 3.51	0.0207*
Weight (kg)	53.68 ± 9.33	56.14 ± 9.95	0.5630
BMI (kg/m^2^)	23.34 ± 3.54	22.96 ± 3.66	0.8872
Body surface area (m^2^)	1.50 ± 0.15	1.56 ± 0.13	0.3452
Rectal temperature (°C)	37.23 ± 0.16	37.05 ± 0.17	0.0232*
Skin temperature (°C)	34.45 ± 0.43	34.01 ± 0.73	0.2354
Systolic blood pressure (mmHg)	130.4 ± 14.19	109.7 ± 16.40	0.0255*
Diastolic blood pressure (mmHg)	74.38 ± 6.05	68.20 ± 9.18	0.0697
Resting mean arterial pressure (mmHg)	92.88 ± 6.45	81.90 ± 11.21	0.0182*
Heart rate (bpm)	72.38 ± 3.96	60.40 ± 7.76	0.0021*
Resting metabolic rate (kcal/min)	0.96 ± 0.29	0.87 ± 0.18	0.7129

*P values are calculated by Mann-Whitney test. All values are expressed as mean ± SD

### Dietary patterns and physical activity scores

External factors, such as dietary habits and lifestyle, can also affect the gut microbiota composition. To investigate the differences in dietary pattern and lifestyle between the inland and island subjects, we assessed habitual diet and physical activity using a 3-day recall dietary assessment and the International Physical Activity Questionnaire (IPAQ), respectively. Total energy intake was not significantly different between the inland and island groups (Table 3). However, the island group had a significantly higher intake of animal lipids compared to the inland group (*P* = 0.0401).

**Table 3:** Average daily dietary intake of the inland and island group subjects

Daily intake of nutrients	Inland (*n* = 7)	Island (*n* = 8)	*P* value*
Total energy (kcal)	1475 ± 346.57	1505.2 ± 343.55	0.7515
Carbohydrate (g)	249.89 ± 54.76	258.83 ± 54.85	0.8354
Protein (g)	55.60 ± 22.24	54.19 ± 10.12	0.6707
Fiber (g)	23.69 ± 7.52	22.80 ± 8.58	0.6005
Lipids (g)	31.43 ± 15.47	30.05 ± 12.24	0.8354
Animal lipid (g)	9.37 ± 4.66	16.1 ± 9.82	0.0401*
Plant lipid (g)	22.06 ± 12.64	13.94 ± 4.14	0.2303
Cholesterol (mg)	180.00 ± 69.43	195.10 ± 50.15	0.9203
Saturated fatty acid (g)	5.69 ± 4.45	6.28 ± 6.07	>0.9999
Monounsaturated fatty acid (g)	6.63 ± 3.40	8.15 ± 6.84	0.8920
Polyunsaturated fatty acid (g)	5.97 ± 2.81	6.30 ± 2.85	0.7515

*P values calculated using Mann-Whitney test. All values are expressed as mean ± SD

**Fig. 1:**
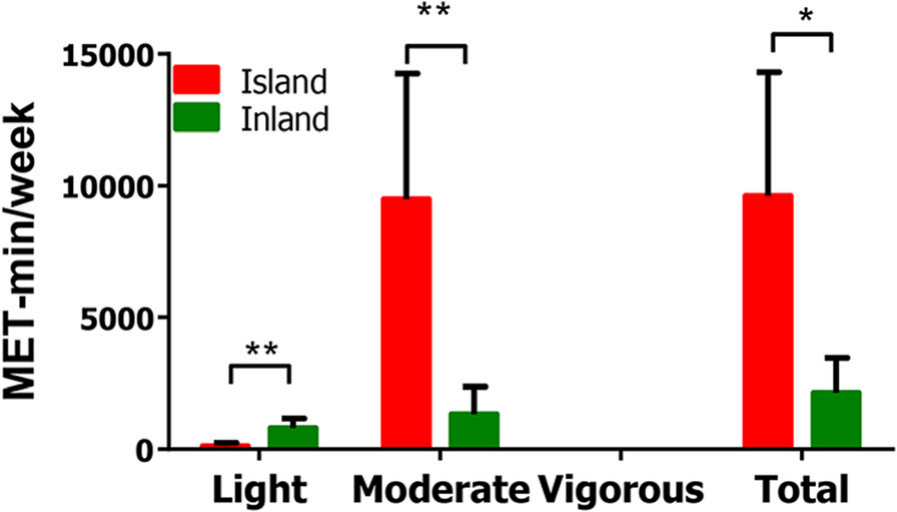
Comparison of the level of physical activity between subjects from island and inland. Physical activity in island and inland subjects as measured by International Physical Activity Questionnaires (IPAQ) [ref IPAQ]; MET-min/week. Data are presented as mean ± SD (*n* = 7–8 per group) and *P* value was estimated by Mann-Whitney test. *P < 0.05, **P < 0.001

Next, we compared the total median weekly energy expenditure of the inland and island groups (Fig. 1). A significant difference was observed in total physical activity between island and inland subjects (*P* = 0.0190), with the island group having a significantly higher total physical activity score than the inland group. Physical activity over 1 week can be divided by the level of intensity. The frequency of light-intensity physical activity, which included walking, was significantly higher in the inland subjects than in the island subjects (*P* = 0.0023). However, the frequency of moderate-intensity physical activity was significantly higher in the island subjects than in the inland subjects. The moderate-intensity physical activities that island subjects preformed most frequently were farming and diving. These results suggest that the subjects in these two regions had different lifestyles. In addition, differences in the regional characteristics of the living area likely influence people’s dietary patterns and lifestyles.

### Microbial community composition

**Fig. 2:**
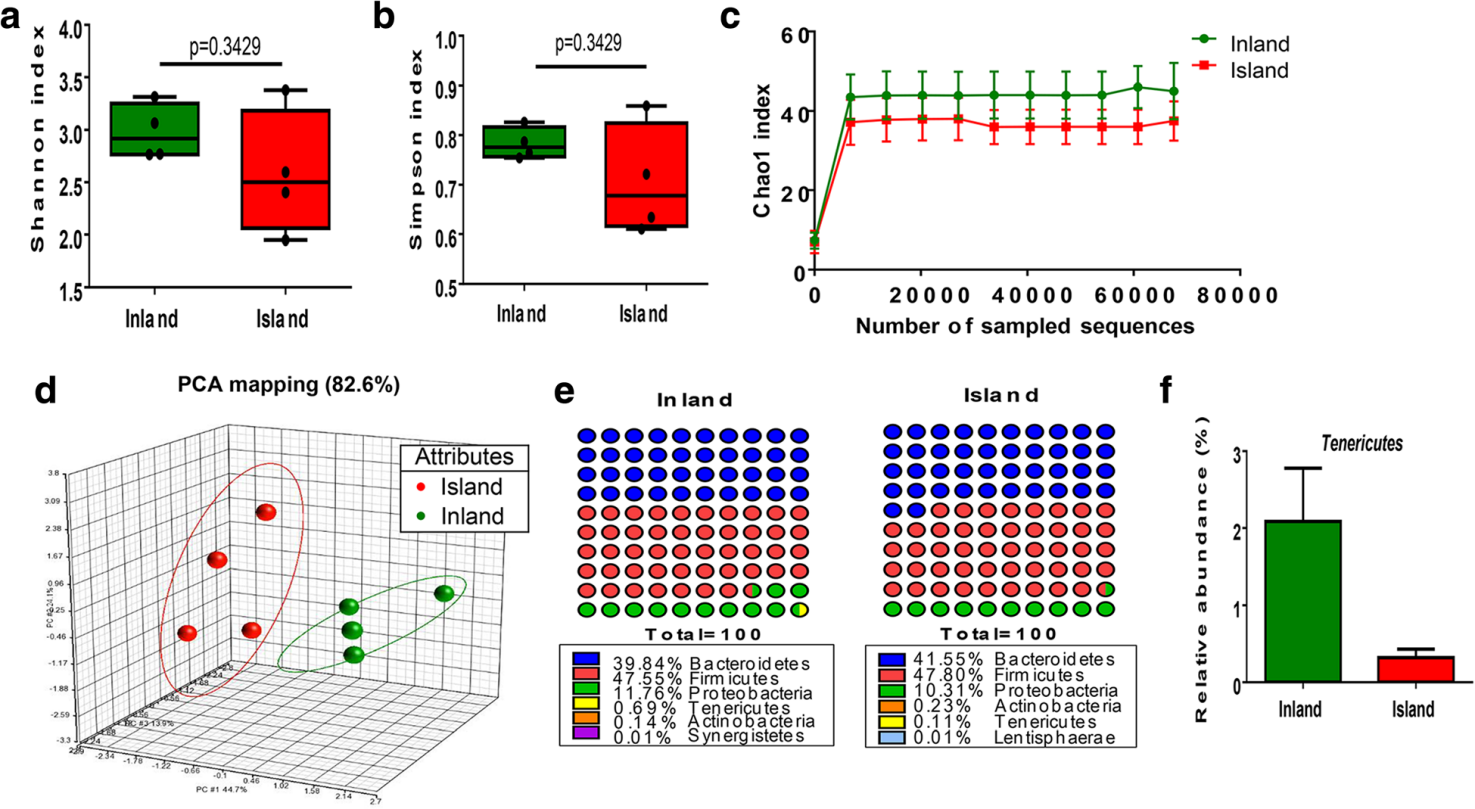
Gut microbial diversity and composition in island and inland subjects. Alpha diversity measures include **a** Shannon, **b** Chao1, and **c** Chao1 index. The interquartile range is shown as a *box* with the median marked as a *horizontal line*, and minimum and maximum from lower and upper quartile represent error bar. *P* values were determined using the Mann-Whitney *U* test (**a**, **b**). Rarefaction curves indicate microbial richness within gut derived from Inland and Island participants. **d** Principal component analysis (PCA) of intestinal bacteria at genus level. Each *color dot* represents data from individual participants (*n* = 4 per group), and multiple dots from the same group are shown as *large circles*. **e** Composition of fecal microbiota at the phylum level in the inland (*n* = 4) and island (*n* = 4) group. Composite bacterial communities are shown as relative abundance (%). **f** The differences in relative abundance of Tenericutes between inland and island group

To investigate the effect of different living environments on the intestinal microbiota composition, the microbiota profiles of feces were evaluated. First, we investigated the entire alpha diversity—including the Shannon, Simpson, and Chao1 indexes—which reflects the diversity and richness of the gut microbial community [19]. The inland subjects tended to have higher Shannon, Simpson, and Chao1 indexes than the island subjects (Fig. 2a–c). Therefore, these data suggest that the inland group tended to have higher bacterial diversity and richness than the island group.

Principal component analysis (PCA) of bacterial communities showed that the island subjects had a bacterial community distinct from that of inland subjects (Fig. 2d). By taxonomy-based analysis, the three most abundant phyla were found to be Bacteroidetes, Firmicutes, and Proteobacteria, which accounted for an average of 99% of the sequences (Fig. 2e). The relative abundance of Bacteroidetes, Firmicutes, and Actinobacteria in the island subjects was higher than that in the inland subjects. However, the average relative abundance of the phylum Proteobacteria in the inland and island subjects was 11.76 and 10.31%, respectively. In addition, Synergistetes was present only in the inland subjects, whereas Lentisphaerae was detected only in the Island subjects. Interestingly, the relative abundance of Tenericutes was lower in the island subjects than in the inland subjects (Fig. 2f). This is in agreement with a previous report of a lower abundance of Tenericutes in mice during cold exposure [20].

**Fig. 3:**
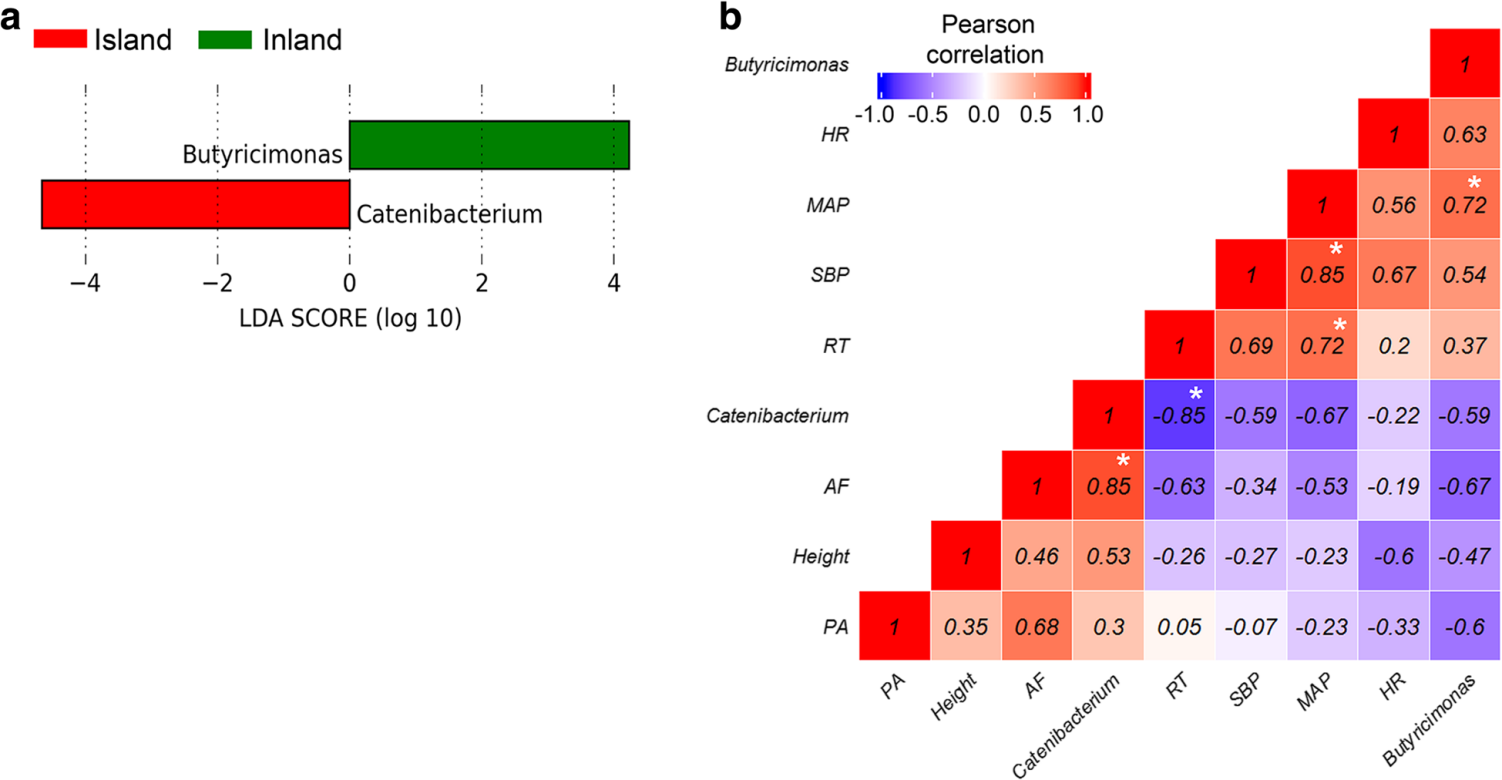
LEfSe identified the most differentially abundant genus between island and inland group. **a** Inland elderly women with enriched taxa are indicated with a positive LDA score (*green*), and taxa enriched in island elderly women have a negative score (*red*). Only taxa meeting an LDA significant threshold >2 are shown. **b** Pearson correlation coefficients among two genera, environmental factors, and host characteristics. Negative correlations are in *blue color* and positive correlations in *red*. Correlations are shown in a *light* (poor correlation) to *dark* (strong correlation) color scale. *Asterisk* represents statistical significance (*P* < 0.05). *HR* heart rate, *MAP* mean arterial pressure, *SBP* systolic blood pressure, *RT* rectal temperature, *AF* amount of animal fat intake, *PA* physical activity

In total, 72 genera were identified. To identify differentially abundant genera between the inland and island subjects, we employed the linear discriminant analysis (LDA) effect size (LEfSe) algorithm [18] using a web-based tool. Seventy-two genera were identified in two groups. We found two significantly enriched genera with a threshold LDA score higher than 2.0. In the island subjects, significant overrepresentation of *Catenibacterium* (*P* < 0.05) was observed. In contrast, the inland subjects were found to have significantly higher levels of *Butyricimonas* (*P* < 0.05; Fig. 3a). Thus, the effects of living environment on subjects’ physiological characteristics were significantly associated with their gut microbiota profiles.

### Correlations among enriched genera, subjects’ characteristics, and subjects’ external factors

Our observation that the inland and island subjects had distinct gut microbiota profiles prompted us to examine the correlation of the relative abundance of genera and characteristics of subjects. We focused on MAP, SBP, RT, average amount of animal fat intake (AF), height, physical activity (PA) score, and HR because these parameters were statistically different between the two groups. We assessed the correlations between the relative abundance of two genera—*Catenibacterium* and *Butyricimonas*—and the seven parameters using the Pearson correlation coefficient (Fig. 3b).

The Pearson correlation coefficient matrix showed a positive correlation between MAP and SBP (*r* = 0.85, *P* < 0.05). In addition, MAP was significantly positively correlated with RT (*r* = 0.72, *P* < 0.05). Further, there was a significant positive correlation between the relative abundance of *Butyricimonas* and MAP (*r* = 0.72, *P* < 0.05). The relative abundance of *Catenibacterium*, which was present mainly in the island subjects, was negatively correlated with RT (*r* = −0.85, *P* < 0.05). Moreover, there was a positive correlation between the relative abundance of *Catenibacterium* and the average amount of AF intake (*r* = 0.85, *P* < 0.05). However, there was no significant correlation between the total amount of physical activity and the relative abundance of *Catenibacterium* and *Butyricimonas*. Collectively, these results imply that lifestyle and physiological characteristics, which are significantly influenced by living environment, may affect gut microbiota profiles.

## Discussion

Recently, there has been growing interest in the human gut microbiota due to its beneficial roles in maintaining health and preventing disease; therefore, identifying the genetic and environmental factors that shape the microbiota has been the focus of this research. The distinct intestinal microbiome profile differs according to lifestyles and/or geography; however, few studies have investigated the relationship between environmental factors and gut microbiome profiles, especially in the elderly. Here, we investigated the correlation of intestinal microbiota profiles with the physiological parameters of the body—temperature and blood pressure and daily lifestyles—dietary pattern, and physical activities in healthy elderly people from island and inland regions of Korea.

Many studies have reported that diet patterns influence the gut microbiota profile. Moreover, diet patterns differ geographically in the same country [21]. Also, a number of dissimilarities in dietary patterns were reported between island and inland areas of South Korea [22]. The current study found that island subjects took in a greater amount of lipids from animal sources compared to inland subjects. In this context, one unanticipated finding was that no differences were found in BMI and weight between island and inland subjects. It may be that island subjects benefited from increased physical activity, which increased their energy expenditure. It is somewhat surprising that no other differences in habitual dietary pattern were observed. A possible explanation for this is that the sample size was insufficient to detect a significant difference in food intake. Therefore, further studies are needed to assess differences in the dietary pattern of elderly women from inland and island areas.

The microbiota sequencing data demonstrate that the main dominant phyla were Firmicutes and Bacteroidetes in both the inland and island subjects. Our findings are consistent with a report that these two phyla were major components of the gut microbiota in Koreans and other ethnicities [23]. Jeju Island is known as “the longevity village” [24]. According to Park et al. [23], elderly persons there have a low abundance of the phylum Tenericutes. This result is in agreement with our finding that the relative abundance of Tenericutes in the island subjects was lower than that in the inland subjects. In addition, the most interesting finding was that the island and inland groups had distinct gut microbiota profiles. The butyric acid-producing genus *Butyricimonas* of the phylum Bacteroidetes was highly abundant in inland subjects [25, 26]. Butyric acid is a short-chain fatty acid and a product of the fermentation of dietary fiber by the gut microbiota [27]. In our study, dietary fiber intake tended to be higher in inland subjects than in island subjects. This may explain in part the increased abundance of butyric acid-producing bacteria in inland subjects. *Catenibacterium* from the Firmicutes phylum was more abundant in island subjects, but their biological function is unclear. However, a high-fat diet caused a significant increment in the abundance of *Catenibacterium mitsuokai* [7]. In addition, the relative abundance of Firmicutes was higher in the obese and those with a high-fat diet [28]. In contrast to earlier findings, however, no evidence of an abnormal BMI was detected in island subjects. However, intake of animal lipids was higher in island subjects than in inland subjects. Although the identified bacteria do not fully represent their populations, our results provide further evidence of the differences in the gut microbiota profiles between individuals residing in island and inland areas.

Another important finding was the significant correlation between two genera and some characteristics of the subjects. Rectal temperature, which was low in island subjects, was negatively correlated with the relative abundance of *Catenibacterium*. This result is in accord with a recent report that continuous cold exposure influences the gut microbiota profile, especially *Akkermansia muciniphila* [20]. In contrast to earlier findings, however, no evidence of elevation of *A. muciniphila* abundance was detected in island subjects. Contrary to expectations, this study did not find a significant correlation between *Catenibacterium* and physical activity. In this study, *Butyricimonas*, a genus of butyric acid-producing bacteria, was enriched in inland subjects, and the relative abundance of this genus was positively correlated with mean arterial pressure. This result is in agreement with Pluznick’s findings, which showed that short-chain fatty acids, especially propionate, can partially modulate the blood pressure in mouse via Olfr78 [29]. It can therefore be assumed that the difference in blood pressure between subjects from the two regions is associated with gut microbiota composition, which is influenced by subjects’ lifestyle and living environment.

Despite these promising results, the current study has several limitations. First, the sample size was small, and so the results may not be representative of the populations of the island and inland areas. This limitation may have attenuated the ability to detect differences in dietary patterns and gut microbiota profiles. Second, it is possible that the subjects may have under- or over-reported their daily intake of food items because of low compliance and old age. Finally, the current study found that the gut microbiota profile is correlated with lifestyle and health characteristics. However, the effect of regional differences on gut microbiota profiles is unknown. A further study of the causal relationship between the natural environment and the human gut microbiota is therefore warranted.

## Conclusions

In conclusion, to our knowledge, this study is the first to analyze comparatively the gut microbiota profiles of elderly women from inland and island areas. These groups of subjects have different dietary patterns, levels of daily activity, lifestyle, and physiological characteristics. The gut microbiota from island subjects was dominated by the bacterial genus *Catenibacterium*, whereas *Butyricimonas* was the most common genus in inland subjects. The relative abundance of *Catenibacterium* was negatively and positively correlated with rectal temperature and amount of dietary animal fat intake, respectively. In addition, the relative abundance of *Butyricimonas* showed a strong positive correlation with mean arterial pressure. Therefore, the present study suggests that geographical differences may be associated with the lifestyle of a population and their gut microbiota composition. Our findings suggest that geographical characteristics should be considered in the same ethnic group, when gut microbiota composition is being analyzed, to provide information on the commensal bacteria profiles of elderly women.

## Abbreviations

AF: Amount of animal fat intake
HR: Heart rate
LDA: Linear discriminant analysis
MAP: Mean arterial pressure
MET: Metabolic equivalent
PA: Physical activity
rRNA: Ribosomal ribonucleic acid
RT: Rectal temperature
SBP: Systolic blood pressure
